# Automated extraction of standardized antibiotic resistance and prescription data from laboratory information systems and electronic health records: a narrative review

**DOI:** 10.3389/frabi.2024.1380380

**Published:** 2024-03-08

**Authors:** Alice Cappello, Ylenia Murgia, Daniele Roberto Giacobbe, Sara Mora, Roberta Gazzarata, Nicola Rosso, Mauro Giacomini, Matteo Bassetti

**Affiliations:** ^1^ Clinica Malattie Infettive, IRCCS Ospedale Policlinico San Martino, Genoa, Italy; ^2^ Department of Informatics, Bioengineering, Robotics and System Engineering (DIBRIS), University of Genoa, Genoa, Italy; ^3^ Department of Health Sciences (DISSAL), University of Genoa, Genoa, Italy; ^4^ UO Information and Communication Technologies (ICT), IRCCS Ospedale Policlinico San Martino, Genoa, Italy; ^5^ Healthropy, Savona, Italy; ^6^ Health Level 7 (HL7) Europe, Brussels, Belgium

**Keywords:** antibiotic resistance, antimicrobial stewardship, automated extraction, EHR, LIS

## Abstract

Antimicrobial resistance in bacteria has been associated with significant morbidity and mortality in hospitalized patients. In the era of big data and of the consequent frequent need for large study populations, manual collection of data for research studies on antimicrobial resistance and antibiotic use has become extremely time-consuming and sometimes impossible to be accomplished by overwhelmed healthcare personnel. In this review, we discuss relevant concepts pertaining to the automated extraction of antibiotic resistance and antibiotic prescription data from laboratory information systems and electronic health records to be used in clinical studies, starting from the currently available literature on the topic. Leveraging automatic extraction and standardization of antimicrobial resistance and antibiotic prescription data is an tremendous opportunity to improve the care of future patients with severe infections caused by multidrug-resistant organisms, and should not be missed.

## Introduction

Antimicrobial resistance in bacteria has been associated with significant morbidity and mortality in hospitalized patients (Courvalin, 2016; Bassetti et al., 2017). Both development of severe infection and treatment of severe infection caused by multidrug-resistant (MDR) bacteria are active fields of clinical research through observational, surveillance studies assessing the epidemiology of MDR organisms, and through either observational studies or randomized controlled trials investigating efficacy and safety of old and new antibiotics for the treatment of MDR infections (Karaiskos et al., 2019; Kanj et al., 2022; Gill et al., 2024).

Collection of data for the studies mentioned above is usually performed manually, through collection of relevant demographics, clinical, therapeutic, microbiological, and prognostic information that is necessary for correctly evaluating epidemiology of MDR organisms and for assessing factors favorably or unfavorably impacting diagnosis or prognosis, by means of appropriate statistical models (Maraolo et al., 2021; Giacobbe et al., 2023a).

However, in the era of big data and of the consequent frequent (although not an absolute rule) need for large study populations, manual collection of data for research studies has become extremely time-consuming and sometimes impossible to be accomplished by overwhelmed healthcare personnel (Giacobbe et al., 2020). Against this backdrop, automated extraction of data from electronic health records (EHRs) and laboratory information systems (LISs) is attracting increasing attention for the possibility of rapidly and automatically collecting the large amounts of data necessary for training and evaluating complex statistical or machine learning models, at the same time relieving healthcare personnel from the difficult (or sometimes impossible) task of manually collecting thousands of variables (Puing et al., 2019). However, the automated extraction should be of high-quality, accurate, reproducible, and standardized, which could prove not so easy tasks, although essential in line with FAIR principles (Findability, Accessibility, Interoperability, and Reusability) (McEwen and Fedorka-Cray, 2002; Huys et al., 2007; Wilkinson et al., 2016).

In the present narrative review, we discuss relevant concepts pertaining to the automated extraction of antibiotic resistance and antibiotic prescription data from LISs and EHRs to be used in clinical studies, starting from the currently available literature on the topic (see **Table 1**).

**Table 1:** Results of the literature search.

Authors	Data Type	Information on the automatic extraction process
Brotherton et al. (Brotherton et al., 2020)	Structured	Not specified
Chao et al. (Chao et al., 2018)	Structured	VigiLanz software
Grundmeier et al. (Grundmeier et al., 2018)	Structured Unstructured	Machine learning classifier models: logistic regression, random forest
Hawes et al. (Hawes et al., 2018)	Unstructured	POLAR
Inglis et al. (Inglis et al., 2021)	Unstructured	Manual, Natural Language Processing
Koller et al. (Koller et al., 2019)	Unstructured	MOMO
Macy et al. (Macy et al., 2021)	Structured	Not specified
Simoes et al. (Simões et al., 2018)	Structured	SQL and Java ETL module
Teodoro et al. (Teodoro et al., 2012)	Structured	Java ETL module
Tunio et al. (Tunio et al., 2023)	Structured	Automatic, Manual
Verberk et al. (Verberk et al., 2023b)	Unstructured	Natural Language Processing
Vermassen et al. (Vermassen et al., 2020)	Unstructured	Natural Language Processing
Yigzaw et al. (Yigzaw et al., 2020)	Structured	SMILe, SQL

POLAR, Population Level Analysis and Reporting; MOMO, Monitoring of Microorganism; SQL, Structured Query Language; ETL, Extract Transform Load; SMILe, Snow Medrave Interaction Library Extension.

## Methods

On November 5, 2023, a literature search was conducted on PubMed using the keywords (EHR OR EHRs OR “electronic health record” OR EMR OR “electronic medical record” OR EPR OR “electronic personal record” OR “laboratory information system*”) AND (antibiotics OR antibiogram OR antibiotic OR antimicrobial OR “antimicrobial resistance” OR “antibiotic stewardship”) AND (“data extraction” OR “data extracted” OR “data retrieval” OR “information extraction” OR “information extracted” OR “information retrieval” OR “data mining”) NOT review and carefully evaluating the references of the articles retrieved. The resulting papers were manually screened by checking their title, abstract and full text. Inclusion criteria were: (i) publication after 2018; (ii) focus on antibiotic resistance or antibiotic prescription data from LISs or EHRs; and (iii) involvement of automatic data extraction from LISs or EHRs. We considered focus on genetic data as the only exclusion criterion. Given the availability of dedicated research in the literature (McEwen and Fedorka-Cray, 2002; Huys et al., 2007), a separate specific paragraph in the manuscript has been dedicated to automated extraction of antibiotic resistance data in veterinary medicine. Eventually, 13 articles were selected for discussion in the present review.

### Data extraction from electronic health records

There are several possible methods to deal with information extraction from datasets, more specifically from EHRs. The wide range of adopted solutions is a direct consequence of the heterogeneity of the data structure: EHRs present a peculiar framework which varies by country and state, and may vary even by hospital within a limited geographic area (Ciampi et al., 2016). Moreover, EHRs often contain both structured and unstructured data, which require different extraction procedures, and may include missing data that need management (Tayefi et al., 2021).

Although different methodologies have increasingly become available for automatic extraction of data, manual extraction is still used much more frequently (Cuningham et al., 2020; Inglis et al., 2021; Tunio et al., 2023). Manual extraction is extremely time-consuming. However, it is applicable to any type and form of data. For this reason, it is frequently preferred to automatic extraction, since automatic extraction algorithms are heavily contingent on the data type and specialized personnel are needed to develop and use them.

Automatic extraction algorithms show substantial differences depending on whether the data handled are structured or unstructured. The automatic extraction of structured data requires a clear understanding of the schema of the sources, fields and relationships within data (Giacomini and Nappo, 2006). Specific queries are necessary to extract specific data. Then, the extracted data may undergo transformation and normalization to adhere to a standard format, and validation checks are implemented to ensure data integrity (e.g., checks for data type consistency and range validation). Against this background, challenges such as incomplete or inaccurate data are also frequently present (Austin et al., 2021). Therefore, the management of both missing values and data redundancies should also better be carefully defined and implemented *a priori*.

Algorithms dedicated to the extraction of information from unstructured data, which refers to data lacking a predefined, organized format like traditional databases, often present a more tangled framework. The absence of a rigid structure requires a more sophisticated approach, involving dedicated algorithms to navigate through the inherent variability and complexity present in unstructured data sources that present a richer and more intricate analytical context. This is because, to deal with data without a definable structure a priori, algorithms must be able to figure out, for example, where to find the information of interest, and this requires a deep understanding of the data (Zaman et al., 2020). For all articles retrieved and included in the present review, the unstructured data type was free text. The discipline that deals with understanding and managing free text data is called Natural Language Processing (NLP), which deals with making the natural language understandable to machines (Chowdhary, 2020). Many steps are often required to perform an appropriate extraction. Usually, the free texts dataset undergoes pre-processing, which involves cleaning, transforming, and organizing raw data to enhance quality and suitability for further processing. Typically, this step is followed by entity recognition and use of machine learning models. Challenges include ambiguity and variability, with variations in approaches (Casey et al., 2021; Reading Turchioe et al., 2022; Zhang et al., 2022).

As mentioned above, depending on the structure of the dataset and the specific data type, various tools can be used for the extraction of data, and different solutions can be adopted. For example, several data management tools were employed for the extraction of antibiotic resistance and antibiotic prescription data in the articles included in the present review, such as VigiLanz software, POLAR, MOMO, ETL (Extract, Transform, Load) module, SMILe and SQL. A more detailed description of these tools is available in **Table 2** (Teodoro et al., 2012; Klass et al., 2013; Chao et al., 2018; Grundmeier et al., 2018; Hawes et al., 2018; Simões et al., 2018; Koller et al., 2019; Pearce et al., 2019; Brotherton et al., 2020; Vermassen et al., 2020; Yigzaw et al., 2020; Macy et al., 2021; Verberk et al., 2023b; VigiLanz, 2023; Medexter Healthcare).

**Table 2:** Automatic extraction tools.

Tool	Description	Limitations	Comments
VigiLanz software	Web-based Clinical Decision Support System (CDSS), queries to extract data from various sources	Complex integration with other systems, constraints related to privacy and security, costs	For the essential tasks of patient identification and automatic data extraction, Chao et al. (2018) employed the capabilities of the VigiLanz software. VigiLanz is a clinical surveillance software and healthcare solution designed both for data extraction and monitoring. It operates as a web-based clinical support tool that can be used both to query healthcare data from multiple sources such as pharmacy and LIS and facilitating the monitoring within healthcare settings (Klass et al., 2013). For the objectives of their study, the authors employed VigiLanz to extract and analyze data from different domains, which include administrative, pharmaceutical and microbiological records.
POLAR	Natural Language Processing applied in free text note	Ambiguous use of words and acronyms, spelling errors in the original free-text, references to real world entities and third persons raising privacy concerns	In the study conducted by Hawes et al. (2018), the authors’ primary purpose was to extract data that is routinely collected from EHRs in general practice. Accordingly, the goal was to use these data to understand and describe how physicians prescribe antibiotics. The authors opted for a software, known as POLAR (Verberk et al., 2023b). This program is designed to convert general practitioners’ clinical text notes from EHRs into Systemized Nomenclature of Medicine-Clinical Terms (SNOMED-CT) codes, adding a layer of semantic richness to the data. At the core of POLAR’s functionality lies its implementation of sophisticated NLP algorithms, designed and used to analyze the grammatical structure of the clinical sentence and test a variety of sentence formations against the SNOMED-CT descriptions.
Custom SQL module	Relational databases management	Limited to relational databases, performance issues	SQL is a domain-specific programming language designed for managing and manipulating relational databases. It provides a standardized way to interact with databases, allowing users to perform tasks such as querying data, updating records, inserting new data, and managing database structures. SQL is used by Database Management Systems (DBMs) to communicate with and manage relational databases, making it an essential tool for developers, data analysts, and database administrators.
MOMO tool	Manage laboratory data	Costs	In the article by Koller et al. (2019), the main goal of the authors was to address and solve identification challenges within the analysis of microbiology results. Their focus was on increasing the accuracy and efficiency of this process by implementing a collaborative approach between human intervention and automation. For the purpose of microbiology data extraction and analysis, the authors used the MOMO tool (Vermassen et al., 2020). MOMO is a microbiological analysis tool with strong clinical features and it can import data from the hospital microbiological LIS and also from EMRs. MOMO systematically evaluates incoming textual identifiers, such as sample details, detection methods, microbes, and antibiotics to align them with existing thesaurus entries. Its main function is to ensure compatibility and provide different analysis options.
Custom Java ETL module	Extract, transform and load tool	Complexity, poor scalability	A Java ETL module is a software component designed to facilitate the extraction of data from source systems, its transformation into a desired format, and the subsequent loading of that data into a target database or data warehouse. The Java ETL module typically leverages Java programming language libraries and frameworks to perform these tasks. It involves defining extraction rules to retrieve data from diverse sources, applying transformation logic to standardize or modify the data as needed, and finally loading the processed data into a destination repository. The extraction phase involves connecting to various data sources such as databases, files, or Application Programming Interfaces (APIs), and pulling the relevant datasets. The module often incorporates error handling mechanisms to manage issues that may arise during the ETL process. This module has been employed for extracting clinical data from different sources (patient data, microbiology laboratory and pharmacy data) in the paper of Simoes et al (Simões et al., 2018). The aim of the paper was the development of HAITooL, a real-time surveillance and clinical decision-support system; to extract the data, a web-based information system was developed to support a Structured Query Language (SQL) Server that extracts and aggregates the different data types. Although not explicitly reported in the text, Teodoro et al. (2012) have graphically described the data extraction process, from which it can be seen that a Java ETL module was used in this case too.
SMILe	Data extraction from different EHRs	Costs	The purpose of the article by Yigzaw et al. (2020) was to present a distributed architecture, designed to provide physicians with feedback on their clinical performance by comparing it with that of their colleagues in different healthcare institutions, and, at the same time, safeguarding the privacy of patients, physicians, and the healthcare institutions involved. A key aspect of this distributed architecture is the ability to monitor antibiotic prescriptions at the group level. Since multiple health facilities are involved, using different systems to collect and store data, the authors needed to find a way to overcome the problem of heterogeneity in these data. Therefore, to address the heterogeneity of EHRs from different healthcare institutions, Yigzaw et al. used the SMILe tool. The SMILe tool, operating within a healthcare facility, daily retrieves data from the local Electronic Health Record system. This data undergoes transformation and loading into the research database. The research database adheres to a standardized data model defined in SQL format.

POLAR, Population Level Analysis and Reporting; SQL, Structured Query Language; MOMO, Monitoring of Microorganism; ETL, Extract Transform Load; SMILe, Snow Medrave Interaction Library Extension.

### The relevance of standard terminologies

To properly perform automatic information extraction, it is crucial to have a standardized data type whenever possible, in order to minimize ambiguity and enable interoperability and operational efficiency (Navigli and Velardi, 2005). In particular, the adoption of Standardized Clinical Terminology emerges as a pivotal element. This standardized terminology provides a common language to describe clinical information, minimizing ambiguity in the data and enabling optimal operational efficiency. According to the World Health Organization (WHO), a Standardized Clinical Terminology is a “compilation of terms used in the clinical assessment, management and care of patients, which includes agreed definitions that adequately represent the knowledge behind these terms and link with a standardized coding and classification system” and “Use of standardized terminology will result in better and safer patient care and more efficient health services” (Executive Board 118, 2006). Some of the papers included in the present narrative review adhere to defined terminology standards, employing a number of standardized languages to codify clinical terminologies and facilitate the automatic extraction process.

The Anatomical Therapeutic Chemical (ATC) Classification System, maintained by the WHO, organizes drugs into a hierarchy with five levels. At the top of the system, there are fourteen main groups, or 1st levels, categorized by anatomy or pharmacology. Each main group is further divided into 2nd levels, which can be either pharmacological or therapeutic groups. The 3rd and 4th levels further refine into chemical, pharmacological or therapeutic subgroups, while the 5th level specifically identifies the chemical substance (WHOCC, 2023). Therefore, since the ATC Classification is an international system used to classify drugs, in the context of the study conducted by Hawes et al. (2018), ATC codes were adopted to categorize antibiotics in a standardized way, thus providing a common basis for the data analysis.

Yigzaw et al. (2020) also employed the use of the ATC Classification System and, in addition, they used the International Classification of Primary Care, 2nd edition (ICPC-2). ICPC-2 is a classification system used to organize patient data and clinical activities in General/Family Medicine and primary care settings. This classification system helps the organization of various aspects, including the reason for the patient encounter, problems or diagnoses addressed, interventions undertaken and the structured arrangement of these data into a framework of episodes of care (WHO, 2003). In their work, Yigzaw et al. (2020) decided to use the ATC and ICPC-2 classification systems to systematically organize the acquired information, diagnosis and medical prescriptions from both the EHRs and the research databases used to store data.

The International Classification of Diseases, 9th Revision (ICD-9) has been developed by WHO with the aim to provide a standardized system to classify injuries, diseases and causes of death (ICD - ICD-9, 2023). The coding structure is represented with up to five digits, similarly to the 10th Revision (ICD-10) which, however, surpasses ICD-9 in greater detail and granularity of coded elements (ICD-10 Version: 2019). The coding structure of this newer revision is alphanumeric code with up to seven characters, enabling better accuracy to accommodate progress in technology and medical knowledge. These Standardized Clinical Terminologies have been exploited in the study of Chao et al. (2018), specifically ICD-10 codes are utilized to define a cohort of healthy individuals, excluding children with particular previous conditions. The ICD-10 codes are used also in the paper of Grundmeier et al. (2018), focusing on the task of switching the coding system from ICD-9 to ICD-10: descriptive words coupled with classification codes are used as input features because of the transition. The study highlights the complexity of accurately mapping between ICD-9 and ICD-10 codes, mainly for conditions where antibiotics are indicated. The one last of the papers analyzed where the ICD standard has been utilized is the study of Vermassen et al. (2020) where ICD-9 codes were used to define which patients had septic shock.

Another Standardized Clinical Terminology which is widely used and established is Systematized Nomenclature of Medicine - Clinical Terms (SNOMED CT), which is a complete clinical terminology developed for use in EHRs and other health information systems. It goes above and beyond coding diseases and procedures to represent clinical constructs and the links between them (SNOMED CT). SNOMED CT is a tree-like hierarchical structure where concepts are represented by univocal numeric codes. It can be employed in order to map and/or normalize various local terminologies and codes, facilitating a univocal understanding of these terms during the extraction process. In their work, Teodoro et al. (2012) discuss the usage of standard terminologies, including SNOMED CT along with WHO’s Anatomical Therapeutic Chemical (WHO-ATC) (WHOCC - ATC/DDD Index, 2023) and Universal Protein Resource (UniProt/NEWT) (UniProt, [[NoYear]]), in the ARTEMIS system. These terminologies have been mapped to the DebugIT Core Ontology and local concepts not covered within them are normalized against them using automatic classification tools.

### Free text management

Dealing with free text, it is not known *a priori* where the information of interest is located. Therefore, in addition to the extraction of natural language fields from the datasets of interest, NLP algorithms are typically implemented to properly use the information, as presented in several articles (Grundmeier et al., 2018; Vermassen et al., 2020; Inglis et al., 2021; Verberk et al., 2023b). EHRs developers are delivering an increasing number of tools, with the aim of supporting surveillance tasks, which usually utilize structured data (Hoffman and Podgurski, 2013), yet these kinds of tools are unable to manage unstructured data (such as free text).

Verberk et al. (2023a) initially described and validated a semi-automated surveillance algorithm for post-operative surgical site infections in multiple hospitals. In another paper (Verberk et al., 2023b), they proposed an improvement of the algorithm, including NLP as an add-on to free text clinical notes. To extract timely information, they implemented a list of keywords, considered as features, which also included the names of the antibiotics. This way, all texts of the clinical notes were compared with the list of keywords and each match was counted.

Vermassen et al. (2020) aimed to use NLP to identify patients with septic shock from clinical text notes from EHRs. The free text fields considered were “*reason for admission*”, “*current medical history”*, “*daily notes*”, “*conclusion of admission*”. Four dictionaries were created to apply NLP. The first dictionary contained only the term “*septic shock*”; the second dictionary contained terms related to infection; the third dictionary contained terms related to the need for vasopressors; the fourth dictionary contained terms related to increased lactate levels. The authors implemented two search strategies: (i) an explicit strategy, which only used the first dictionary, so patients were labeled as suffering from septic shock if this term was found in the text fields of the medical record; (ii) a combined strategy, where all four dictionaries were used and patients were labeled with septic shock if the explicit term was retrieved or if a dictionary-matching term for infection, vasopressors, or lactate was retrieved.

The aim of the study from Inglis et al. (2021) was to define machine learning models able to classify penicillin adverse drug events (ADRs) and evaluate the risk of true allergy, utilizing the free-text fields of EHRs. Once these fields were retrieved, the information about prescriptions and other crucial features was manually extracted.

### Clinical data heterogeneity

Antibiotic data encompass a wide range of information, including but not limited to classes of antibiotics, mechanisms of action, resistance patterns, and efficacy of different antibiotics against specific pathogens. Understanding the complexities of antibiotics is crucial to make informed decisions in clinical practices and public health interventions. All but one of the papers contained antibiotic-related data, and all but one included general clinical data, while the other data types are strictly dependent on the specific purpose of the paper in question.

### Automatic extraction in veterinary medicine

The threat of antibiotic resistance in the medical domain cannot be completely separated from that in the veterinary one. As widely demonstrated in numerous studies (Guardabassi et al., 2004; Lloyd, 2007; Allen et al., 2010; Graveland et al., 2010), animals have the ability to acquire and transmit MDR pathogens. This constitutes a potential channel for the exchange of antimicrobial resistance (AMR) with humans. The articles by Hur et al. (2022) and by Tharmakulasingam et al. (2023) explored the automatic extraction of antibiotic data in the veterinary field. It is worth noting that these studies focused deeply on automatic extraction, explaining in detail the tools created specifically for this purpose.

The study by Hur et al. (2022) aimed to describe the use, dose and common indications of antibiotic use, using NLP techniques to extract and analyze the information present in clinical records. In particular, NLP was applied to the free text fields of clinical notes, with the aim of extracting the relevant information mentioned above. The authors employed state-of-the-art NLP models, specifically VetBert (Hur et al., 2020) [the veterinary adaptation of ClinicalBert (Huang et al., 2020)]. This transformer model identified the reason for administering antimicrobials directly from the free text fields. By blending the information from structured data with the information extracted from the free-text fields through the above NLP techniques, the authors were able to calculate dose, duration, and indication for antimicrobial use.

The article by Tharmakulasingam et al. (2023) illustrates a novel way to predict development of AMR in bacteria. They used a 1D-Transformer, which helped to understand information about antibiotic use. A peculiarity of this model was indeed that an attempt was made to explain its predictions. The authors thus employed an explainable Transformer model, attempting to explain the model’s decisions in order to be understood and interpreted. Explainability (whenever completely interpretable models cannot be used or are less accurate in predictions) could be crucial, especially in medical fields, since trust in the model’s suggestions is essential for adoption by healthcare professionals. The article suggests using explainable AI (XAI) techniques, such as the Multi-Baseline Integrated Gradient approach, to enhance understanding of how the model reached its predictions and make it more transparent to users.

## Discussion

The automation of data extraction can strongly benefit from the possibility of standardization of health information systems as it could efficiently use the data communication structures between hospital systems and those of the Health Information Infrastructure (Ozaydin et al., 2020). First of all, it is necessary that the tools used for extraction are as unobtrusive as possible, often based on services that allow the exchange of data even between tools from technically different platforms (Gazzarata and Giacomini, 2016). However, it is not sufficient for there to be a technically effective possibility of transmitting data between different systems, it is essential that there is an understanding of the information at a higher level, therefore the use of technical interoperability tools, such as Fast Healthcare Interoperability Resources (FHIR) messages and similar (Wulff et al., 2021; Duda et al., 2022), but also knowledge sharing systems at a higher level (Blobel et al., 2023).

One of the key points to achieve a correct reuse of the data automatically extracted from health information systems is the adequate management of terminology. It was seen in previous sections how, in almost all projects taken into consideration in this review, standardized vocabularies were correctly used to manage the project’s internal terminology. However, at the same time it was clearly seen that the choice of vocabularies used is quite wide, due to the legitimate choice on the part of the specific communities that carried out each project to use that terminological collection that they consider most efficient for their purpose. Therefore, for a correct comparison between the results of individual projects, we believe it is extremely important to use the mapping services of international bodies such as the Unified Medical Language System (UMLS) centered in the USA https://www.nlm.nih.gov/research/umls/index.html, or the similar initiative set up in Europe such as Athena (https://athena.ohdsi.org/search-terms/start). However, these mappings must be maintained over time because the different vocabularies are frequently updated. Terminological services developed on international standards or machine learning technologies can be useful in these operations (Gazzarata et al., 2017; Kang et al., 2021).

Finally, the ability to create large, accurate, and standardized datasets of automatically extracted data concerning both antibiotic susceptibility (in bacteria causing the infection) and antibiotic use (in humans with the infection) should be coupled with improvements in the automatic extraction of other clinical features (e.g., information related to the acute phase of the disease, baseline comorbidities, other concomitant infectious and non-infectious conditions) (Giacobbe et al., 2023b; Giacobbe et al., 2023c). In turn, standardization of this process would allow to exploit the aid of either classical statistical models or up to date machine learning algorithms for accurately investigate clinically relevant associations between features and selected outcome of interests (e.g., diagnosis of a specific antimicrobial resistant infection, prognosis of a specific antimicrobial resistant infection), thereby improving identification of factors able to improve either diagnosis of treatment. Creating such large and accurate datasets through classical manual collection is likely nearly (or totally) impossible in the current big data era. Consequently, improving automatic extraction and standardization of antimicrobial resistance and antibiotic data is a tremendous opportunity for improving care of future patients with severe infections caused by MDR organisms, and should not be missed.

## Author contributions

AC: Writing – original draft, Writing – review & editing. YM: Writing – original draft, Writing – review & editing. DG: Conceptualization, Writing – original draft, Writing – review & editing. SM: Conceptualization, Writing – review & editing. RG: Supervision, Writing – review & editing. NR: Supervision, Writing – review & editing. MG: Supervision, Writing – review & editing. MB: Supervision, Writing – review & editing.
